# Analytical Methods for the Determination of Fatty Acid Esters of Hydroxy Fatty Acids (FAHFAs) in Biological Samples, Plants and Foods

**DOI:** 10.3390/biom10081092

**Published:** 2020-07-22

**Authors:** Maroula G. Kokotou

**Affiliations:** Chemical Laboratories, Department of Food Science and Human Nutrition, Agricultural University of Athens, Iera Odos 75, 11855 Athens, Greece; mkokotou@aua.gr; Tel.: +30-2105294261

**Keywords:** anti-diabetic, anti-inflammatory, fatty acid esters of hydroxy fatty acids (FAHFAs), lipidomics, liquid chromatography–mass spectrometry (LC-MS)

## Abstract

Fatty acid esters of hydroxy fatty acids (FAHFAs) constitute a class of recently identified novel lipids exhibiting anti-diabetic and anti-inflammatory effects. Due to their high biological significance, a tremendous effort has been devoted to the development of analytical methods for the detection and quantitation of FAHFAs during the last five years. The analysis of FAHFAs is very challenging due to the great number of possible regio-isomers arising from the great number of possible combinations of FAs with HFAs, and the low abundancies of FAHFAs in biological samples. The aim of this review article is to summarize all the cutting-edge analytical methodologies for the determination of FAHFAs in biological samples, plant tissues and food matrices, with emphasis on extraction and analysis steps. All the analytical methodologies rely on the use of liquid chromatography–mass spectrometry (LC-MS), providing high sensitivity due to the MS detection. Powerful and robust analytical methodologies may highly contribute in studying FAHFAs levels under various biomedical conditions, and facilitate our understanding of the role of these lipid species in physiological and pathological conditions.

## 1. Introduction

During the last two decades, a variety of endogenously generated lipids have been recognized as biomolecules that dynamically affect human physiology and pathophysiology [1]. In 2014, Kahn, Saghatelian and co-workers reported the discovery of a previously unrecognized class of endogenous mammalian lipids exhibiting anti-diabetic and anti-inflammatory effects [2]. Employing a quantitative mass spectrometry (MS) lipidomics approach, they compared a big set of ions in adipose-selective overexpression of Glut4 (AG4OX) and wild type (WT) mice and they observed a cluster of ions in AG4OX mice that was elevated >16-fold. The measured accurate mass of these ions enabled them to calculate the molecular formulas, indicating that all were members of a single lipid class containing a unique signature of four oxygen atoms. However, such formulas did not correspond to any metabolite known at that time in metabolite databases, including LIPIDMAPS [3]. Fragmentation studies suggested that these lipid species consisted of hydroxy fatty acids (HFAs) acylated by fatty acids, and thus these lipids were named as fatty acid esters of hydroxy fatty acids (FAHFAs). Continuing with a targeted MS approach, they indeed identified in mouse serum 16 FAHFA family members that consisted of four fatty acids and four hydroxy fatty acids in different combinations [2].

**Figure 1:**
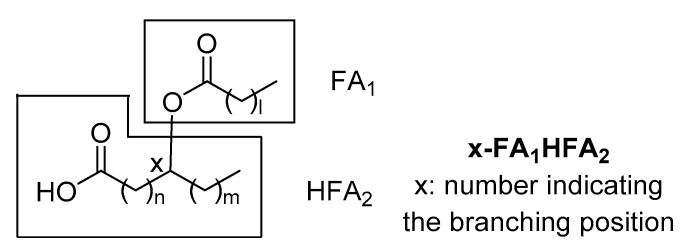
General structure of fatty acid esters of hydroxy fatty acids (FAHFAs).

Since that time, several branched FAHFA families have been identified in serum, mammal tissues, plants and foods. Each one consists of multiple regio-isomers, in which the hydroxy group participating in the ester bond is at different positions (e.g., 5- or 9-). The general structure of FAHFAs is depicted in Figure 1. It should be noticed that in 1982, Nicolaidis and Ruth reported in human and steer meibum an unusual group of high molecular weight fatty acid derivatives, which were identified as (O-acyl)-ω-hydroxy fatty acids [4]. These lipids differ from those identified and studied during the last six years, because the ester group is at the ω-position, making them linear and not branched.

During the last five years, a tremendous effort has been devoted to the development of analytical methods for the detection and quantitation of FAHFAs in biological samples as well as in plants and foods. The great number of possible combinations of FAs with HFAs together with the great number of possible regio-isomers makes the analysis of FAHFAs very challenging due to overlapping of the various regio-isomers in the liquid chromatography (LC) conditions. The aim of this review article is to summarize and discuss the analytical methodologies developed so far, which all employ liquid chromatography–tandem mass spectrometry (LC-MS/MS).

## 2. Diversity of FAHFAs and Elucidation of Their Structures

### 2.1. Diversity of FAHFAs

**Figure 2:**
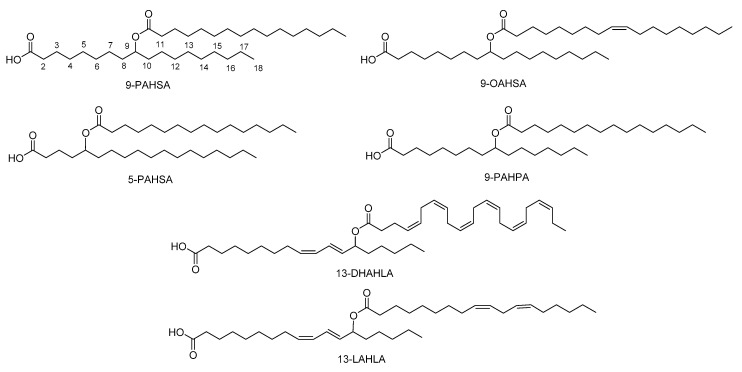
Representative structures of FAHFAs.

The major FAHFAs that have been detected are a combination of palmitic acid (PA), stearic acid (SA), oleic acid (OA), or palmitoleic acid (PO) with their corresponding hydroxy fatty acids (HPA, HSA, HOA, HPO respectively), providing PAHPA, OAHPA, PAHOA, OAHOA, PAHSA and OAHSA [2,5] (Figure 2). A variety of regio-isomers have been identified with the ester linkage at position C5, C7, C8, C9, C10, C11, C12 and C13. Although most of the FAHFAs contain saturated lipid chains, also polyunsaturated FA-derived FAHFAs have been uncovered, including omega-3 polyunsaturated-derived and linoleic-derived FAHFAs [6,7]. Samples of the major FAHFAs are commercially available by various companies. In addition, methodologies for their synthesis, either in racemic or chiral form, have been developed [2,8,9,10,11].

### 2.2. Mass Spectrometry Studies for the Structure Elucidation of FAHFAs

**Figure 3:**
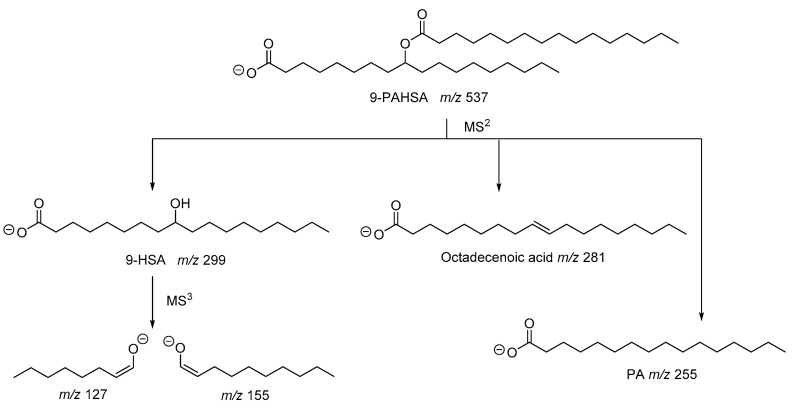
Fragmentation pathway of 9-PAHSA.

As described in the seminal work of Kahn and coworkers, the structure of PAHSA was elucidated by mass spectrometry studies [2]. The fragmentation of the precursor ion observed at *m*/*z* 537 generated ions with *m*/*z* 255, 281 and 299, which were attributed to palmitic acid (PA), octadecenoic acid and hydroxystearic acid (HSA), respectively (Figure 3). Since the ion with *m*/*z* 537 did not contain any double bond, octadecenoic acid resulted from fragmentation in the MS and, thus, PAHSA was proposed as an ester between PA and HSA. The ions observed at *m*/*z* 127 and 155, also recorded in an MS^3^ experiment [6], indicated the position of hydroxyl group at C-9 of stearic acid (Figure 3).

In 2015, α computer-generated library with 3267 tandem mass spectra (MS/MS) for 1089 FAHFA species was presented [12]. In silico electrospray ionization (ESI) MS/MS spectra in negative ion mode were modelled based on the reference spectra of 9-PAHSA obtained under 10, 20 and 40 V collision-induced dissociation (CID) voltages acquired with ultra-high performance liquid chromatography-quadrupole time-of-flight (UHPLC-QTOF) MS/MS profiling methods at 4 spectra/s. To generate the fragments with *m*/*z* 127 and 155, indicating the position of the hydroxyl group, MS/MS experiments with a longer acquisition time of 1 spectrum/s were required at a higher voltage (40 V).

Determination of the ester position in isomeric FAHFAs was studied by ion trap mass spectrometry [13]. The CID mass spectra of precursor [M–H]^−^ ions for 5-PAHSA, 9-PAHSA, 12-PAHSA and 18-PAHSA showed the same three predominant product ions in varying ratios. However, the MS^3^ spectra acquired from 5-HSA, 9-HSA, 12-HSA and 18-HSA revealed distinct diagnostic product ions unique to each isomer in the region below *m*/*z* 240, allowing for pinpointing of the ester location.

**Figure 4:**
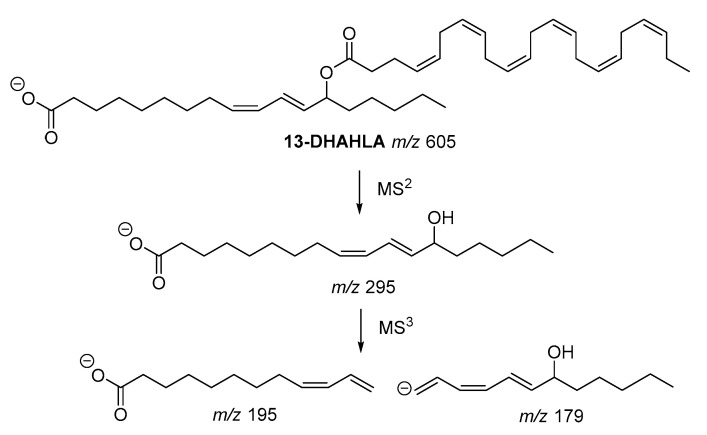
Fragmentation pathway of 13-DHAHLA.

The fragmentation pathway of the polyunsaturated 13-DHAHLA is shown is Figure 4. The precursor ion (*m*/*z* 605) generated the fragment with *m*/*z* 295, which subsequently gave rise to fragments with *m*/*z* 195 and *m*/*z* 179, indicating the position of hydroxyl group [6]. Similar fragments were produced from the fragmentation of 13-LAHLA [7].

MS studies play a pivotal role in the elucidation of the structure of these natural lipids. In particular, MS^n^ fragmentation studies allow the determination of the branching position, discriminating the various positional isomers.

## 3. Sample Preparation

### 3.1. Methods for the Extraction of FAHFAS and Solid Phase Extraction (SPE) Protocols

FAHFAs have been studied in a variety of matrices. Biological samples, plant tissues and various foods have been investigated to explore the existence of FAHFAs and to estimate their contents. The sample preparation procedure in general consisted of two steps: solvent extraction and solid phase extraction (SPE). Saghatelian and co-workers applied [5], either in sera or in tissues, the widely used general protocol (Bligh–Dyer method [14]), which is employed for the extraction of common lipid classes (e.g., fatty acids, phospholipids, sphingolipids, acylglycerols, cholesterol and cholesterol esters) and relies on a 1:1:2 mixture of aqueous buffer:methanol:chloroform, slightly modified. This extraction method has found application in the majority of the published analytical studies. Hu et al. described a three-phase solvent system of methyl *tert*-butyl ether (MTBE), methanol and water for the total lipid extraction [15], which was an adaptation of the original method described for lipid extraction by MTBE [16].

SPE enables the enrichment of FAHFAs, while at the same time removes other metabolites and contaminants that could cause signal suppression and/or impair chromatographic resolution. Saghatelian and co-workers described in detail the analytical-scale SPE protocol [5], which was based on the hexane:ethyl acetate flash chromatography method used during the synthesis of PAHSA standards [2]. The silica extraction cartridge is first washed with hexane, followed by 95:5 hexane:ethyl acetate to remove neutral lipids, such as triacylglycerols and cholesterol esters. Then, FAHFAs are eluted with ethyl acetate and the samples resulting from this procedure can be reconstituted in methanol [5]. Two years later, the same group described a faster protocol for the endogenous FAHFA measurements [17], employing positive pressure (nitrogen) to push solvents though a Strata SI-1 silica SPE cartridge (500 mg silica, 3 mL, Phenomenex, Torrance, CA, USA). By this modification, they shortened the SPE step time from 4 h to 1 h. Other methods described the use of a Hysphere C8 catridge [18], a HyperSep silica SPE column (500 mg, 6 mL, Thermo Scientific, Waltham, MA, USA) [15,19,20,21], or a strong anion exchange (SAX) SPE-cartridge (1 mL, 50 mg, Weltech, Wuhan, China) [22,23,24]. 

### 3.2. Derivatization Procedures

The low abundancies of FAHFAs in biological samples, plants and foods make their detection very challenging. In addition, it is known that fatty acids and similar carboxylic acids normally show low ionization efficiencies in ESI source in negative mode. The detection sensitivity of free carboxylic acid compounds could be drastically enhanced in ESI-MS analysis through derivatization, aiming at the introduction of a fixed charge site (e.g., a tertiary amine) [25,26,27,28].

**Figure 5:**
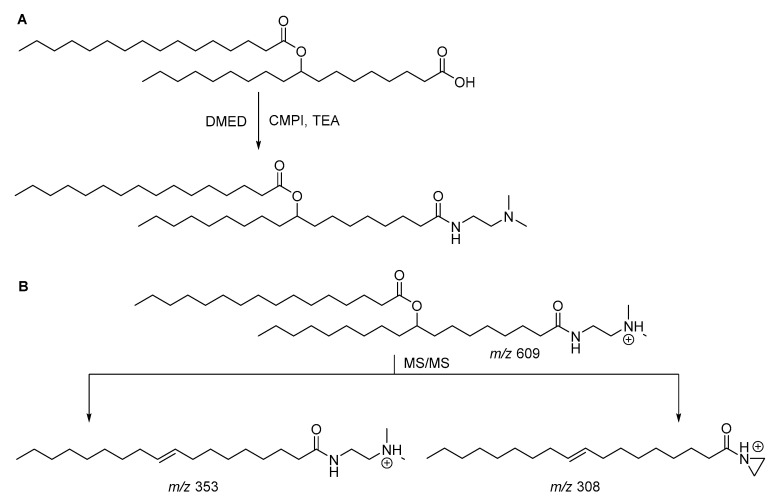
2-Dimethylaminoethylamine (DMED) derivatization reaction (**A**) and fragmentation of DMED-labeled 9-PAHSA (**B**).

Based on their previous studies [27,28], Feng and co-workers used a pair of isotope labeling reagents, 2-dimethylaminoethylamine (DMED) and *d*_4_-DMED to label the purified samples and standard FAHFAs, respectively [22]. FAHFAs were converted to the corresponding amides using 2-chloro-1-methylpyridinium iodide (CMPI) as the coupling reagent in the presence of triethylamine (Figure 5A). The fragmentation of DMED-labeled 9-PAHSA is shown in Figure 5B, as an example. The fragmentation of the DMED-labeled 9-PAHSA precursor ion (*m*/*z* 609) generated in positive ionization mode by tandem MS two characteristic product ions at *m*/*z* 308 and 353. It mainly occurred in the amine C-N bond and ester C-O bond through elimination of the dimethylamine from DMED and palmitic acid from PAHSA. Upon DMED labeling, the detection sensitivities of FAHFAs increased and the limits of detections (LODs) of labeled FAHFAs ranged from 0.01 to 0.14 pg. This derivatization approach has been used by the same group in their more recent studies [23,24].

**Figure 6:**
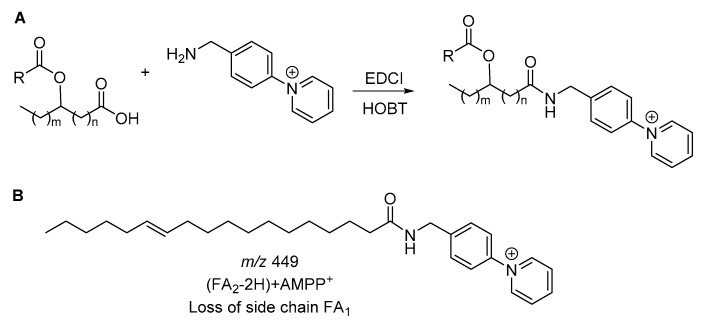
*N*-(4-Aminomethylphenyl) pyridinium (AMPP) derivatization reaction (**A**) and representative structure of the most abundant ion (**B**).

Most recently, Han and co-workers reported the sensitive analysis of FAHFAs in biological lipid extracts by shotgun lipidomics after one-step derivatization with *N*-(4-aminomethylphenyl) pyridinium (AMPP) [21]. AMPP is one of the most representative derivatization reagents used for charge reversal of carboxylic acids [26]. FAHFA species may be coupled with AMPP using [3-(dimethylamino) propyl]-ethylcarbodiimide hydrochloride (EDCI) as the coupling reagent in the presence of 1-hydroxybenzotriazole (HOBT) (Figure 6A). In positive ionization mode, the most abundant fragment ions in the product-ion MS spectrum of AMPP-derivatized FAHFAs were [(FA_2_-2H) + AMPP] ^+^ ions generated from the neutral losses of side aliphatic chains (Figure 6B).

The choice of the appropriate extraction solvent or mixture of solvents is a decisive factor ensuring the quantitative and unbiased recovery of lipid species from the analyzed biological samples, plant tissues or foods. Although the majority of studies on FAHFAs employ ESI in a negative mode, charge reversal of FAHFAs by appropriate derivatization may considerably increase the sensitivity of the determination.

## 4. Analytical Methodologies

### 4.1. Instrumentation

All the analytical methodologies, which have been employed for performing FAHFA studies, are presented in Table 1, where details on the analytical techniques, the instruments and the columns used as well as the sample preparation procedures are summarized. All the analytical platforms rely on the use of MS detection providing high sensitivity. LC-MS allows versatility due to the availability of selection of (i) chromatographic columns with a variety of stationary phases in combination with appropriate mobile phases to achieve efficient compound separation and (ii) different instrumental configurations (i.e., different ESI charge mode and mass analyzers).

FAHFA spectra are recorded in ESI negative mode, since they contain a free carboxyl group. Positive mode is employed when a derivatization step is performed before the analysis [21,22,23,24]. Triple quadrupole (tandem) mass spectrometers have been mainly employed in FAHFA analysis (Table 1). Triple quadrupole linear ion trap (QTRAP) [6,19], as well as high resolution instruments, such as orbitrap mass analyzer [24] or QTOF [20], have been also employed.

Various reversed phase columns, such as Luna C18, C18 Mediterranea, Acquity UPLC BEH C18 and Kinetex C18, have been used (Table 1). An important issue for biological studies is the elucidation of the configuration of the branching carbon in FAHFAs. Among 6 different chiral columns (Cellulose-1, Cellulose-2, Cellulose-3, Cellulose-4, Amylose-2, and Amylose-1), which were explored using 3 different mobile phases (water/acetonitrile, hexane/ethanol, and methanol/isopropanol), Lux 5 μm Cellulose-3 column was proved the most efficient for the resolution of 9-PAHSA enantiomers [9]. Using this column in combination with an isocratic MeOH/H_2_O/formic acid (96:4:0.1) mobile phase, a clear peak separation of the enantiomers was observed. This method allowed identifying *R*-9-PAHSA as the main source of the elevated 9-PAHSA levels observed in AG4OX perigonadal white adipose tissue (WAT) [9]. Kuda and co-workers were able to separate both regio-isomers and enantiomers from biological samples using an amylose tris(3,5-dimethylphenylcarbamate) column [19]. This column separated the regio-isomers in the order from 5- to 13-PAHSA, while the *R*-enantiomers of PAHSAs were eluted before the *S*-enantiomers. For the majority of PAHSA regio-isomers, the analysis of adipose tissue samples indicated the presence of both *R*- and *S*-enantiomers, while higher levels of *R*-enantiomers were found in human milk samples.

Most recently, a novel method for the analysis of FAHFAs has been presented based on the principles of multi-dimensional mass spectrometry-based shotgun lipidomics [21] and avoiding chromatography. This approach possesses marked sensitivity, high specificity and a broad linear dynamic range of over 3 orders without obvious matrix effects.

### 4.2. Occurrence and Contents of FAHFAs in Biological Samples, Plants and Foods

Multiple reaction monitoring (MRM) mode has been mainly utilized for quantitation purposes. For PAHSA, the *m*/*z* 537→255 transition yielded the highest ion count in all PAHSA standards tested, although relative ratios of detected transitions depend on isomer position [5]. Thus, this is the most reliable choice as the quantifier transition [2,5,6,7]. The quantitative transitions (*m*/*z*) together with the qualifier ions (*m*/*z*) for PAHSA, SAHSA, OAHSA, POHSA, DHAHLA and DHAHDHA are summarized in Table 2. 

A variety of isotopic internal standards have been employed for the quantification of FAHFAs. Zhang et al. compared the use of deuterated PAHSA standards and ^13^C labeled standards [5]. It was observed that heavily deuterated PAHSA standards, for example *d*_31_-PAHSA, showed a substantial forward retention time shift relative to endogenous FAHFAs. As a consequence, they strongly recommend the use of ^13^C labeled standards when retention time alignment is required for the identification of the isomer [5]. ^13^C_4_-9-PAHSA is a commercially available standard which has found wide use [6,15,17,19]. Other labeled standards, such as *d*_4_-12-PAHSA [21], ^13^C_16_-5-PAHSA and ^13^C_16_-9-PAHSA [5], have been synthesized according to standard synthetic techniques.

FAHFAs have been detected and quantified in a variety of sources. The contents of some FAHFAs in various sources (approximate mean values) are presented in Table 3. In the initial studies, serum PAHSAs were measured in human and mice [2,6] and tissue PAHSAs (WAT, brown adipose tissue (BAT), liver, kidney and pancreas tissues) in mice [2].

Another study showed that among eleven FAHFAs detected in serum, PAHSA and PAHOA were the most concentrated FAHFAs in serum, while PAHPA and POHPO were found to present significant differences among different glycemic states of the individuals studied [18]. Further studies revealed the presence of 5-, 6-, 7-, 8-, 9-, 10-, 11- and 13/12-PAHSA regio-isomers in human plasma samples, while 8-, 9-, 10-, 11- and 13/12-OAHSA regio-isomers were also identified, with 8-, 9- and 10-OAHSA having the highest levels [5,17]. Additionally, 9-, 10-, 11-, 12- and 13-OAHSA were found in perigonadal WAT from WT and AG4OX mice, with 9-OAHSA being the mostly highly upregulated OAHSA regio-isomer in AG4OX mice [17]. 

Since dysregulation of WAT function in obesity contributes to the development of insulin resistance and type-2 diabetes, special attention has been paid to the estimation of FAHFA levels in WAT. The contents of FAHFAs in rat WAT were successfully measured and the mean values are presented in Table 4. As shown, 13-PAHSA, 12-PAHSA, 9-PAHSA, 9-OAHSA, 13-SAHSA, 12-SAHSA and 9-SAHSA were quantified, while 5-PAHSA was found lower than the limit of quantification (LOQ) and 13-OAHSA, 12-OAHSA, 5-OAHSA, 5-SAHSA, 13-POHSA, 12-POHSA, 9-POHSA and 5-POHSA were not detected [22]. In addition, the contents and distribution of FAHFAs in rat lung, kidney, thymus, liver and heart tissues were determined, showing that 7 FAHFAs (13-, 12-, 9-, 5-PAHSA, 13-, 12- and 9-SAHSA) were observed in different tissues of rat. Furthermore, the above 7 FAHFAs were detected in human serum samples and 13-PAHSA, 9-PAHSA, 13-SAHSA and 12-SAHSA were found remarkably decreased in serum from human breast cancer patients [22]. Another detailed study of FAHFAs in WAT from golden hamsters identified the existence of sixty-four FAHFAs, belonging to 17 different families [15].

The contents of 5-PAHSA, 9-PAHSA and 13/12-PAHSA were measured in apple, broccoli, beef, chicken, egg yolk and egg white, as described in the first report on the discovery of FAHFAs [2] (Table 3). Last year, a variety of foods have been studied to estimate FAHFA levels [20]. The overall FAHFA mass fraction, expressed in 10^−7^ g/g of plant tissue fresh weight, is shown in Table 5 for whole grain oat, clementine, garlic, pineapple, strawberries, mango, carrot and parsley root. SAHSA was estimated as the most abundant FAHFA in these foods (4.61–9.45 × 10^−8^ g/g fresh weight, mostly present in garlic, clementine, mango, pineapple and onion), followed by SAHOA (2.82–5.89 × 10^−8^ g/g fresh weight, mostly present in garlic, clementine, pineapple, pepper and onion) and PAHPA (2.13–4.59 × 10^−8^ g/g fresh weight, mostly present in carrot, mango, banana, whole grain oat and apple).

A comprehensive screening of FAHFAs using Chemical Isotope Labeling-Assisted LC-MS (CIL-LC-MS) in MRM mode allowed a dramatic improvement of the detection sensitivity and selectivity of FAHFAs by selecting paired-peaks with defined mass differences for MRM scanning mode [23]. In the same work, a strategy for identifying the ester position of FAHFA regio-isomers was proposed, which was based on the characteristic chromatographic retention behaviors on a C18 column. Such an approach allows the identification of regio-isomers when commercial standards are not available or when the analytes are not included in databases. Applying this methodology in plant tissues, 49 FAHFA families were detected in rice and *Arabidopsis thaliana*, including 262 regio-isomers, among which 71 regio-isomers from 11 FAHFA families were further confirmed based on commercially available standards and chromatographic retention behaviors.

Most recently, a DMED-FAHFA in-silico library containing 4290 high-resolution tandem mass spectra covering 264 different FAHFA classes was constructed [24]. This in-silico library is compatible with library search software NIST (National Institute of Standards and Technology) MS Search and the LC-MS/MS data processing tool MS-DIA and thus can be useful to profile novel or yet unrecognized FAHFA lipids in plant or diverse other matrices. An application of the in-silico library in *Arabidopsis thaliana* samples enabled the annotation of 19 DMED-FAHFAs from 16 families, including 4 novel compounds. This approach largely decreased the false positive annotation rate in comparison to low resolution LC-MS/MS.

Targeted LC-MS studies using commercially available reference compounds permit the accurate quantification of FAHFAs in various natural sources. As the technology evolves, new available instruments permit detection of previously undetectable species and quantification at lower levels with impressive precision. On the other hand, untargeted approaches focus on acquiring data for as many species as possible, annotating metabolites and reviewing both known and unknown metabolic changes. Such untargeted approaches may unravel new FAHFA families, or even related species, and expand our knowledge on the existence and metabolic changes of natural FAHFAs. Thus, new opportunities for advancing the FAHFA field constitute a challenge for researchers.

## 5. Biological Relevance

In 2014, the initial report of Kahn and co-workers described the anti-diabetic and the anti-inflammatory activities of FAHFAs [2]. It has been shown that PAHSA levels are reduced in adipose tissue and serum of insulin-resistant humans and are well correlated with insulin sensitivity [2,29]. PAHSAs were also found to reduce inflammatory cytokine production from immune cells and ameliorate adipose inflammation in obesity [2,29]. In addition, PAHSA treatment was shown to regulate innate and adaptive immune responses to prevent mucosal damage and to protect against colitis [30]. Polyunsaturated 13-DHAHLA was demonstrated to exert anti-inflammatory and pro-resolving properties, reducing the macrophage activation by lipopolysaccharides and enhancing the phagocytosis of zymosan particles [6]. Other polyunsaturated FAHFAs were found in plants (for example, in oat oil) and 13-LAHLA suppressed lipopolysaccharide-stimulated secretion of cytokines and expression of pro-inflammatory genes [7]. In 2018, Syed et al. showed that chronic PAHSA treatment improved glucose homeostasis in chow- and high-fat diet fed mice and the GPR40 receptor was identified as a novel PAHSA target [31]. Pflimlin et al. challenged the finding that PAHSAs may improve glucose control in mice [10], however, methodological issues in studying PAHSA biology may mask PAHSA effects [32], complicating the complexity of PAHSA research [33]. Kuda et al. have shown that the Nrf2-mediated antioxidant defense and peroxiredoxin 6 are linked to biosynthesis of 9-PAHSA and that FAHFAs may link the activity of this system with insulin sensitivity in peripheral tissues [34]. A recent in vivo study has shown that PAHSAs attenuate immune responses and promote β-cell survival in autoimmune diabetic mice [35]. A very recent study demonstrates that exercise training induces insulin-sensitizing PAHSAs in adipose tissue of elderly women, highlighting the metabolic benefits of exercise [36]. In healthy mice, high intake of 9-PAHPA or 9-OAHPA increased basal metabolism, but it had no direct effect on body weight [37]. Surprisingly, both studied FAHFAs induced hepatic steatosis and fibrosis in some mice, which were more marked with 9-PAHPA. Again, in mice, dietary triacylglycerol-rich oils of marine origin were identified as optimal nutrients for the induction 13-DHAHLA exhibiting anti-inflammatory properties [38].

## 6. Conclusion

The identification of novel lipid molecules exhibiting bioactivities is of great interest, and this field of research constantly increases our knowledge on human, animal and plant lipidome. The discovery of branched FAHFAs in 2014 is a great example indicating that a variety of previously unrecognized species exist in natural sources, awaiting their identification and the investigation of their bioactivities. FAHFAs constitute a class of lipids different from the classes of usual lipid molecules in terms of their structure. They consist of a hydroxylated fatty acid, which is acylated by another fatty acid. Such a structure leads to a great diversification due to the great number of possible combinations of acyl chains. As a consequence, powerful analytical techniques are required for the identification of such complex lipid classes.

The analysis of FAHFAs is very challenging for two reasons: (a) the great number of possible combinations of FAs with HFAs along with the great number of possible regio-isomers and (b) their low abundancies in natural sources. First, efficient chromatographic methods must be able to ensure the separation of the various regio-isomers in the LC conditions, avoiding the overlapping of the various peaks. Second, highly sensitive detectors able to detect and quantify very low concentrations of FAHFAs are required. All the analytical platforms developed for the analysis of FAHFAs rely on the use of MS detection providing high sensitivity. As summarized in Table 1, various instrumental configurations have been employed so far, allowing the identification of a high number of saturated and polyunsaturated FAHFAs. Although the majority of methods employ an ESI source in a negative mode, the derivatization of FAHFAs in order to achieve charge reversal and to increase the sensitivity of the method has been successfully demonstrated in a number of reports, using an ESI source in a positive mode. All the methods described up to now employ a LC separation of the compounds studied. However, most recently, a shotgun lipidomics approach has been reported [21] possessing marked sensitivity and high specificity.

The methods developed so far permitted the study of endogenous FAHFAs in a variety of human and animal tissues, including WAT and BAT, liver, lung, kidney, thymus and heart tissues, as well as in serum and breast milk. The studies have been also extended in plant tissues from rice and *Arabidopsis thaliana*, while recently, a variety of foods such as oat, apple, clementine, lemon, strawberry, blueberry, mango, kiwi, avocado, pineapple, banana, onion, garlic, cherry tomato, carrot, parsley root, pepper and radish have been studied. 

FAHFAs exhibit both anti-diabetic and anti-inflammatory properties and thus may play an essential role in human health. Thus, analytical methodologies, either targeted or untargeted, may highly contribute in studying FAHFA levels under various biomedical conditions, and facilitate our understanding of the role of these lipid species in physiological and pathological conditions. 

## Figures and Tables

**Table 1:** Overview of the analytical methodologies for the study of FAHFAs.

Analytical Technique	Derivatization	Instrumental Analysis	Column/Mobile Phase	Sample	Sample Preparation— Solvent Extraction/ Cartridge-column	Ref.
LC-MS/MS, (-) ESI mode	No	Agilent 6410 (triple quadrupole) combined with HPLC Agilent 1200 (Agilent, Santa Clara, CA, USA).	Luna C18 column (3 μm, 100 Å, 250 × 2.0 mm)/93:7 methanol:water, 0.01% ammonium hydroxide, 5 mM ammonium acetate; flow rate: 0.2 mL/min for 120 min; temperature: 25 °C	Human or mouse serum, subcutaneous WAT, mouse perigonadal WAT, BAT and liver	Bligh–Dyer method/HyperSep silica SPE column (500 mg bed weight, 6 mL column volume, Thermo Scientific)	[5]
LC-MS/MS, (-) ESI mode	No	QTRAP 5500/SelexION, (Sciex, Framingham, MA, USA). hybrid triple quadrupole linear ion trap mass spectrometer equipped with an ion mobility cell (Sciex, Framingham, MA, USA) combined with LC Ultimate 3000 RSLC (Thermo)	Kinetex C18 (1.7 μm 2.1 × 150 mm column)/ 70% water, 30% acetonitrile (ACN), 0.01% acetic acid, pH 4 (Solvent A) and 50% ACN, 50% IPA (solvent B); flow rate: 0.2 mL/min for 60 min; temperature: 50 °C	Epididymal WAT, subcutaneous WAT, liver, interscapular BAT and serum	Citric acid buffer, methanol, dichloromethane (1:1:2)/Strata SI-1 silica SPE cartridge (50 μg silica, 70 Å, Sigma-Aldrich)	[6]
LC-MS/MS, (-) ESI mode	No	Agilent 6460 (triple quadrupole) combined with HPLC Agilent 1200	C18 Mediterranea column (10 × 4.6 mm, 3 μm)/5 mM ammonium acetate and 0.01% ammonium hydroxide in 98:2 methanol:water (organic mobile phase) and 5 mM ammonium acetate and 0.01% ammonium hydroxide in 98:2 water:methanol (aqueous phase); flow rate: 0.8 mL/min for 20 min; temperature: 25 °C	Human serum	Deproteinization by addition of methanol/On-line SPE Hysphere C8 cartridges (7 mm, 10 × 2.0 mm, Spark Holland, Emmen, Holland)	[18]
UHPLC-MS/MS, (+) ESI mode	DMED and *d*_4_-DMED labeling	Shimadzu MS-8040 triple quadrupole combined with Shimadzu LC-30AD UPLC system (Shimadzu, Kyoto, Japan).	Acquity UPLC BEH C18 column (2.1 × 50 mm, 1.7 μm, Waters)/mobile phase (A) formic acid in ACN/water (0.1%, 6/4, *v*/*v*) and (B) formic acid in IPA/ACN (0.1%, 9/1, *v*/*v*; flow rate: 0.4 mL/min for 22 min; temperature: 40 °C	Rat WAT, lung, kidney, thymus, liver and heart tissues. Human serum from healthy individuals and breast cancer patients	ACN containing 0.1% NH_3_.H_2_O/Strong anion exchange solid phase extraction SAX SPE-cartridge (1 mL, 50 mg, Weltech Co)	[22]
UPLC-MS/MS, (-) ESI mode	No	QTRAP 5500/SelexION, hybrid triple quadrupole linear ion trap mass spectrometer equipped with an ion mobility cell (Sciex) combined with UPLC Ultimate 3000 RSLC (Thermo)	Kinetex C18 (1.7 μm 2.1 × 150 mm column)/70% water, 30% ACN, 0.01% acetic acid, pH 4 (Solvent A) and 50% ACN, 50% IPA (solvent B); flow rate: 0.2 mL/min for 60 min; temperature: 50 °C	Murine tissues and human breast milk	Citric acid buffer, methanol, dichloromethane (1:1:2)/HyperSep SPE column (500 mg/10 mL, 40–60 μm, 70 Å, Thermo)	[19]
UPLC-MS/MS, (-) ESI mode	No	Agilent 6470 triple quadrupole mass spectrometer or Agilent 6550 qTOF combined with an Agilent 1290 UPLC system	Acquity BEH C18 column (100 mm × 2.1 mm, 1.7 μm)/Solvent A: water containing 5 mM ammonium acetate. Solvent B: acetonitrile/2-propanol (3:1, *v*/*v*) containing 2 mM ammonium acetate; flow rate: 0.4 mL/min for 28 min; temperature: 50 °C	WAT from hamsters	Methyl *tert*-butyl ether, methanol, water (5:1.5:1.5)/HyperSep silica cartridge (Thermo Scientific)	[15]
LC-MS, (-) ESI mode	No	TSQ Quantiva LC-MS instrument (Thermo Fisher Scientific)	Acquity UPLC BEH C18 column (1.7 μm, 2.1 mm × 100 mm, Waters)/isocratic 93:7 methanol/water with 5 mM ammonium acetate and 0.03% ammonium hydroxide (*v*/*v*); flow rate: 0.2 mL/min for 30 min; temperature: 25 °C	Perigonadal WAT and human plasma	Phospate-buffered saline (PBS), methanol, chloroform (1:1:2)/Strata SI-1 silica SPE cartridge (500 mg silica, 3 mL, Phenomenex)	[17]
UHPLC-MS/MS, (+) ESI mode	DMED	Shimadzu MS-8045 mass spectrometer combined with a Shimadzu LC-30AD HPLC system	Acquity UPLC BEH C18 column (2.1 mm × 50 mm, 1.7 μm, Waters)/Formic acid in water (0.1%, *v*/*v*, solvent A) and ACN (solvent B); flow rate: 0.4 mL/min for 55 min; temperature: 40 °C	Rice and *Arabidopsis thaliana*	Bligh-Dyer method/Strong anion exchange solid phase extraction (SAX SPE) cartridge (3 mL, 200 mg, Weltech Co)	[23]
LC-MS/MS, (-) ESI mode	No	qTOF Synapt G2-Si mass spectrometer (Waters, Milford, MA, USA) coupled to Waters nanoAcquity UPLC	XTerra MSC18 3.5 μm NanoEase column (75 μm × 150 mm, Waters)/Isocratic elution 93:7 methanol:water phase buffer consisted of 5 mmol/L ammonium acetate and 0.01% ammonium hydroxide; flow rate: 0.7 μL/min for 30 min	Oat (whole grain, coarse flakes and fine flakes), apple, clementine, lemon, strawberry, blueberry, mango, kiwi, avocado, pineapple, banana, onion, garlic, cherry tomato, carrot, parsley root, pepper and radish	Citric acid buffer, methanol, chloroform (1:1.5:3)/HyperSep silica cartridge (500 mg bed weight, 6 mL, Thermo Scientific)	[20]
MS, (+) ESI mode, shotgun lipidomics	AMPP	TSQ Quantiva triple quadrupole mass spectrometer (Thermo Fisher Scientific) equipped with an automated nanospray device (i.e., Nanomate HD, Advion Bioscience, Ithaca, NY, USA)	-	Liver and WAT from homozygous diabetic (*db/db*) and WT mice, and human plasma	HyperSep silica SPE cartridge (200 mg, 3 mL, Thermo Scientific)	[21]
LC-HRMS/MS, (+) ESI mode	DMED	LTQ Orbitrap Elite mass spectrometer (Thermo Fisher Scientific) combined with UltiMate 3000 UHPLC System (Thermo Fisher Scientific).	Acquity UPLC BEH C18 column (2.1 mm × 50 mm, 1.7 μm, Waters)/A ACN/water (6/4, *v*/*v*) containing 0.1% formic acid and B IPA/ACN (9/1, *v*/*v*) containing 0.1% formic acid; flow rate: 0.4 mL/min for 37 min; temperature: 40 °C	*Arabidopsis thaliana*	Methanol, chloroform, water (1:2:1)/Strong anion exchange solid phase extraction SAX-SPE cartridge (200 mg, 3 mL, Weltech Co)	[24]

**Table 2:** Multiple reaction monitoring (MRM) transition list.

Analyte	Quantitative Transition (*m*/*z*)	Qualitative Ions (*m*/*z*)	Ref.
PAHSA	537 → 255	299, 281	[2,5,6,15,17,18]
SAHSA	565 → 283	299, 281	[2,5,15,18]
OAHSA	563 → 281	299, 281	[2,5,15,17,18]
POHSA	535 → 253	299, 281	[2,5,15,18]
DHAHLA	605 → 327	295, 277	[6]
DHAHDHA	653 → 327	343, 325	[6]

**Table 3:** Contents of particular PAHSAs in biological and food samples [2,6].

Source	5-PAHSA (pmol/g)	9-PAHSA (pmol/g)	13/12-PAHSA (pmol/g)	Total PAHSA (pmol/g)
Serum	0.2–0.5 *	1–4 *	2–3 *	7–10 *
WAT	40	100	25	150–200
BAT	180	120	30	250–300
Liver	0	20	10	30
Kidney	5	20	2	-
Pancreas	0	5	4	-
Apple	0.1	0.4	0.8	-
Broccoli	-	1.7	1.3	-
Beef	-	4	6	-
Chicken	0.25	1.2	2	-
Egg yolk	-	4	7	-
Egg white	-	0.3	0.5	-

* pmol/mL.

**Table 4:** Contents of FAHFAs in the WAT of rats [22].

FAHFA	Rat WAT (pg/g)
13-PAHSA	84.6
12-PAHSA	22.1
9-PAHSA	89.8
9-OAHSA	13.9
13-SAHSA	81.9
12-SAHSA	132.1
9-SAHSA	51.7

**Table 5:** Contents of FAHFAs in foods [20].

Source	FAHFAs (10^−7^ g/g Fresh Weight)
Whole grain oat	3.20
Clementine	2.51
Garlic	2.43
Pineapple	2.16
Strawberries	1.59
Mango	1.51
Carrot	1.40
Parsley root	1.14
